# The ZmRCP-1 promoter of maize provides root tip specific expression of transgenes in plantain

**DOI:** 10.1186/s40709-016-0041-z

**Published:** 2016-03-29

**Authors:** Stephen O. Onyango, Hugh Roderick, Jaindra N. Tripathi, Richard Collins, Howard J. Atkinson, Richard O. Oduor, Leena Tripathi

**Affiliations:** International Institute of Tropical Agriculture (IITA), P.O. Box 7878, Kampala, Uganda; Biotechnology and Biochemistry Department, Kenyatta University, P.O. Box 43844, Nairobi, Kenya; Centre for Plant Sciences, University of Leeds, Leeds, LS2 9JT UK

**Keywords:** Plantain, Root specific promoter, Nematode invasion, β-*glucuronidase*, Transgenic defence

## Abstract

**Background:**

Bananas and plantains (*Musa* spp.) provide 25 % of the food energy requirements for more than 100 million people in Africa. Plant parasitic nematodes cause severe losses to the crop due to lack of control options. The sterile nature of *Musa* spp. hampers conventional breeding but makes the crop suitable for genetic engineering. A constitutively expressed synthetic peptide in transgenic plantain has provided resistance against nematodes. Previous work with the peptide in potato plants indicates that targeting expression to the root tip improves the efficacy of the defence mechanism. However, a promoter that will provide root tip specific expression of transgenes in a monocot plant, such as plantain, is not currently available. Here, we report the cloning and evaluation of the maize root cap-specific protein-1 (ZmRCP-1) promoter for root tip targeted expression of transgenes that provide a defence against plant parasitic nematodes in transgenic plantain.

**Results:**

Our findings indicate that the maize ZmRCP-1 promoter delivers expression of β-*glucuronidase* (*gus*A) gene in roots but not in leaves of transgenic plantains. In mature old roots, expression of *gus*A gene driven by ZmRCP-1 becomes limited to the root cap. Invasion by the nematode *Radopholus similis* does not modify Root Cap-specific Protein-1 promoter activity.

**Conclusions:**

Root cap-specific protein-1 promoter from maize can provide targeted expression of transgene for nematode resistance in transgenic plantain.

## Background

Bananas and plantains (*Musa* spp.) are ranked 8th in the global harvest of staple crops [1, 2] providing 25 % of the food energy requirements of over 100 million Africans [3]. Several species of plant parasitic nematodes including *Radopholus similis* cause severe yield losses to these crops [4–6] and there are very few control options available to farmers in Africa. Plantains are not readily improved by conventional plant breeding because of their sterile and triploid nature. This characteristic, however, eliminates the risk of transgene flow and so enhances the biosafety of genetic engineering. Consequently, a transgenic approach that can address the need to control the pests and diseases that hamper production of the crop is favourable [7].

One promising approach for nematode control involves transgenic expression of a non-lethal synthetic disulphide-constrained 7-mer peptide with the amino acid sequence CTTMHPRLC [8]. It is taken up by nematode chemoreceptive neurons and subsequently disrupts coordinated responses to chemoreception and limits root invasion by the pathogen [9, 10]. It confers resistance in the field to a cyst nematode on potato [11] and to *R. similis* on plantain in a glasshouse [8] and field [12], when expressed constitutively with a cellular export signal. It is rapidly degraded in soil and is without adverse effects on non-targeted soil nematodes [8, 11].

Targeted expression for defence such as the synthetic peptide requires a promoter that is active at the site of invasion by the nematodes. In potato, the *Arabidopsis* root cap specific MDK4-20 promoter driving a synthetic peptide conferred a higher level of resistance to *Globodera pallida* than the constitutive CaMV35S promoter [13]. To date, only constitutive heterologous promoters from maize ubiquitin 1, the rice actin 1 [14] or a CaMV35S promoter enhanced for monocot expression [15, 16] have been used for the production of transgenic banana plants. Several promoters are known to be actively driving expression of transgene in the roots of rice including rolC [17], RCg2 [18], Tub-1 [19] and PHT1 [20]. Though rolC and RCg2 also drove *gusA* expression in leaf sheaths, *gusA* expression from the Tub-1 promoter rapidly declined as the plants aged while the expression from the PHT1 promoter is dependent on the level of phosphate in the growing medium. None of the studied promoters provide root tip specific expression required for anti-nematode defences.

The delivering of gene expression to tissues invaded by nematodes by a promoter that drives a defence must also continue to express throughout the infection process. The *gus*A and *gfp* expression from the CaMV35S promoter has been shown to be progressively down regulated at the infection sites of *Meloidogyne incognita* and cyst nematodes in *Arabidopsis thaliana* roots [21, 22]. GUS activity from the CaMV35S promoter is also limited following *Heterodera schachtii* infection in the roots of *A. thaliana* [23]. However, MDK4-20 promoter of *A. thaliana* directs effective root-specific transgenic expression of the secreted nematode-repellent peptide in *A. thaliana* and *Solanum tuberosum* [13]. The *Zea mays* Root Cap-specific Protein-1 (ZmRCP-1) is a homologue of the *Arabidopsis* MDK4-20 gene and is active in lateral root cap cells in maize [24]. In this study, we demonstrate that the ZmRCP-1 promoter provides a root tip specific activity suitable to deliver the anti-nematode defences in a monocot crop plant.

## Results

### Preparation and validation of plasmid constructs

**Fig. 1:**
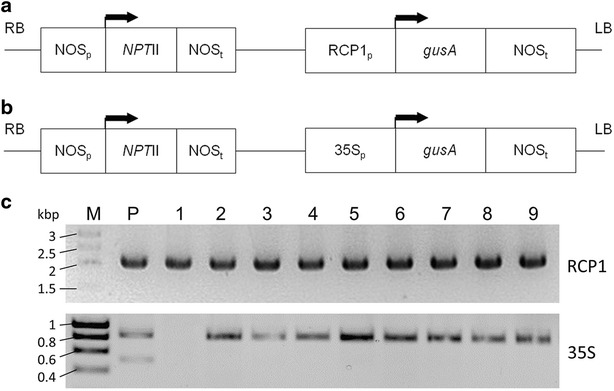
Schematic presentation and validation of plasmid constructs. **a** pBI-RCP-1:GUS- the maize Root Cap-specific Protein-1 promoter (RCP1_p_) drives expression of the *β*-*glucuronidase* (*gus*A) gene. **b** pBI121- the cauliflower mosaic virus CaMV35S promoter (35S_p_) drives expression of the *gus*A gene. For both constructs the nopaline synthase promoter (NOSp) was used to drive expression of the *neomycin phosphotransferase* II (*npt*II) gene and the nopaline synthase terminator (NOSt) was used as the 3΄ poly-A signal for both the selectable marker *npt*II and *gus*A genes. *Left* (LB) and *right* (RB) borders of the T-DNA and translation start sites with the direction of transcription are also indicated. **c** Polymerase chain reaction screen of *Agrobacterium tumefaciens* strain EHA105 colonies (1–9) transformed with pBI-RCP-1:GUS or pBI121. RCP1 primers amplify a 2 kbp fragment and the 35S primers amplify an 835 bp fragment. M—molecular size marker with sizes of bands indicated; P—positive control using the plasmid used for transformation

The construct pBI-RCP-1:GUS was prepared by cloning the ZmRCP-1 promoter and inserting the promoter fragment into the *Hin*dIII and *Bam*HI sites of the binary vector pBI121 immediately 5′ to the *β*-*glucuronidase* (*gusA*) gene (Fig. 1a). The pBI121 plasmid containing the *gusA* gene under regulation of CaMV35S promoter was used as the constitutive expression construct (Fig. 1b). These constructs were confirmed by sequencing and then transferred to *Agrobacterium tumefaciens* strain EHA105. The plasmid constructs were isolated from colonies of transformed *A. tumefaciens* and verified by PCR analysis using the promoter specific primers. The amplicon of 2 kbp was obtained for pBI-RCP-1:GUS amplifying the ZmRCP-1 promoter and fragment of 835 bp amplifying the CaMV35S promoter was observed in pBI121 plasmid construct (Fig. 1c).

### Generation and validation of transgenic plantains

**Fig. 2:**
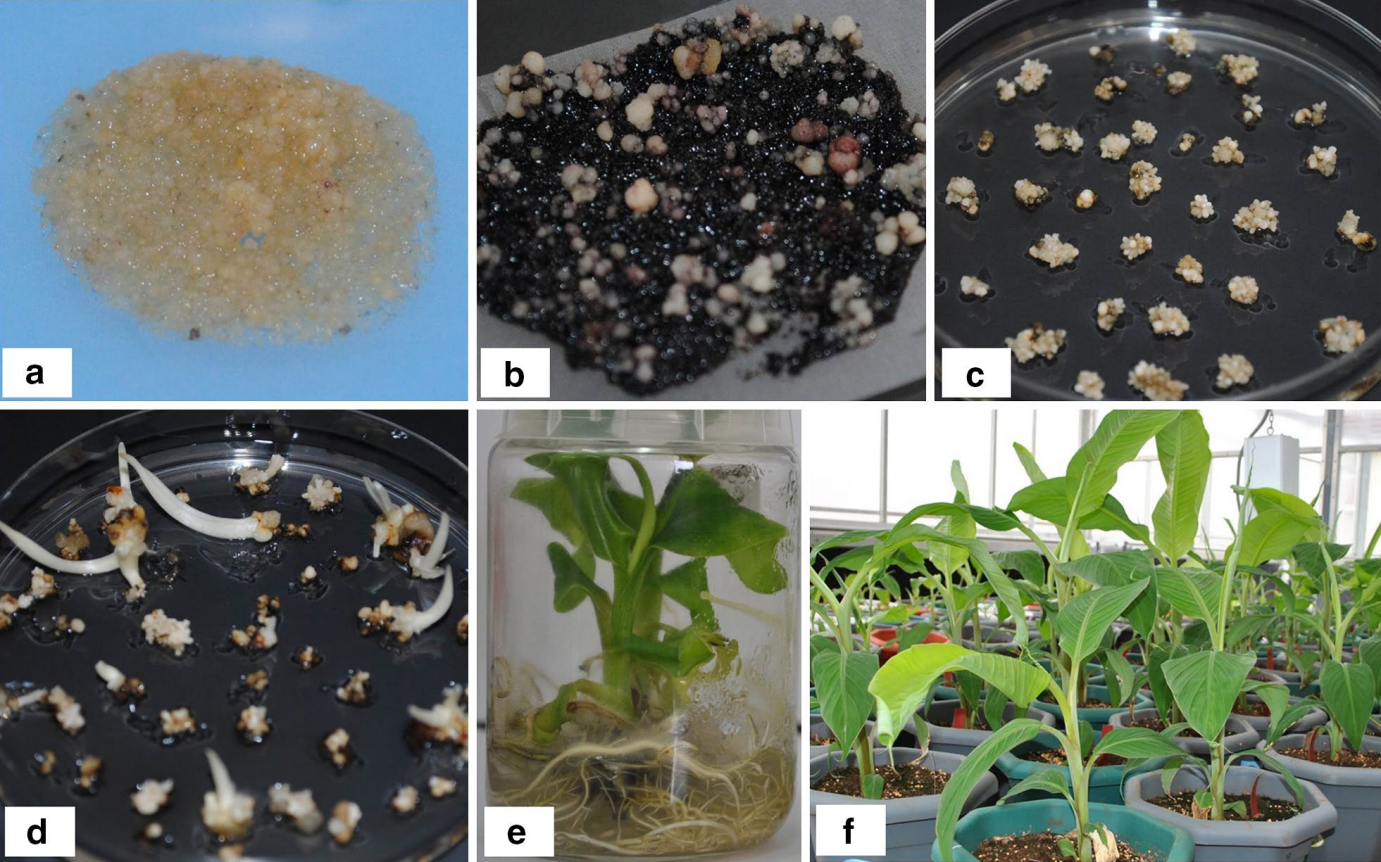
Regeneration of transgenic lines of plantain cv. ‘Gonja manjaya’. **a** Embryogenic cells infected with EHA105 *Agrobacterium tumefaciens* cells harbouring pBI-RCP-1:GUS or pBI121 construct. **b** Proliferation of *Agrobacterium*-infected cells on kanamycin selective medium. **c** Embryos developing on kanamycin selective medium. **d** Germinating transgenic seedlings from mature embryos on selective medium. **e** Transgenic plantlets on rooting medium. **f** Transgenic plants in pots in the glasshouse

**Fig. 3:**
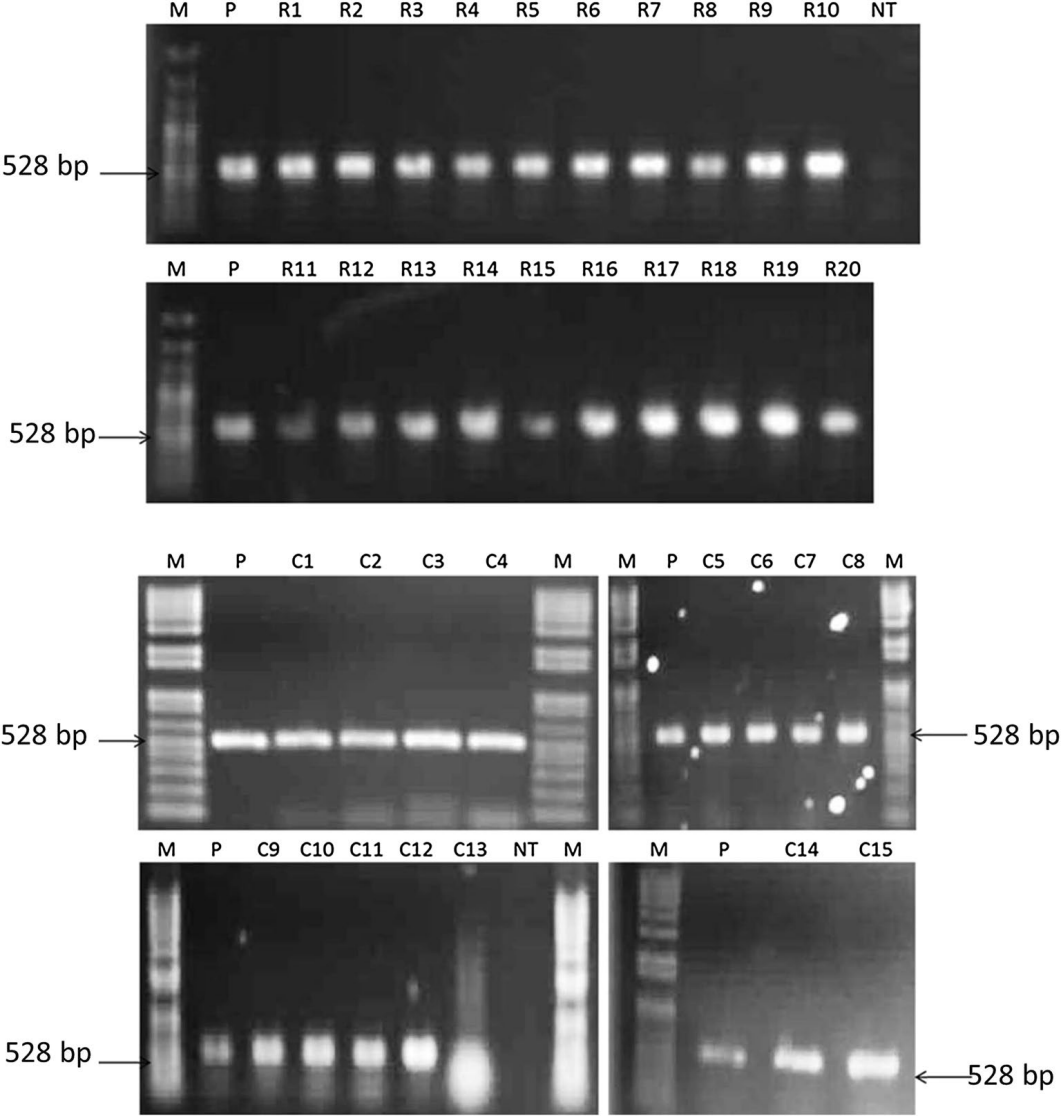
PCR analysis of transgenic plantain lines. Positive transgenic lines screened by PCR for a 528 bp region of the *gusA* gene. Amplification from the pBI121 plasmid was used as a positive control (*P*) and amplification from the genomic DNA of a non-transformed plantain was used as a negative control (*NT*). Twenty RCP-1:GUS lines (R) and 15 35S:GUS lines (C) were positive. 100 bp plus DNA ladder (M) used to assess size of amplified bands

The *Agrobacterium*-infected embryogenic cells multiplied and regenerated on kanamycin selective medium whereas the control untransformed cells turned black (Fig. 2a–d). In total, 20 lines of RCP-1:GUS and 15 lines of CaMV35S:GUS of plantain cv. ‘Gonja manjaya’ were generated from one *Agrobacterium*-mediated transformation experiment using cell suspension. The regenerated transgenic shoots were proliferated and transferred to rooting medium. All the shoots developed roots within 2–3 weeks (Fig. 2e). The rooted plantlets were transferred to the soil in pots in contained glasshouse (Fig. 2f). The lines were confirmed by PCR analysis to contain the *gus*A gene, including those used for further studies (Fig. 3).

**Fig. 4:**
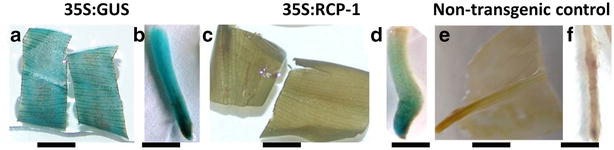
Histochemical GUS assay of leaves and roots of young transgenic lines of plantain cultivar ‘Gonja manjaya’. **a** Leaf of a 35S:GUS line. **b** Root tip of a 35S:GUS line. **c** Leaf of a RCP-1:GUS line. **d** Root tip of a RCP-1:GUS line. **e** Leaf of a non-transgenic control **f** Root tip of a non-transgenic control. *Scale bar* 5 mm

A histochemical GUS assay performed on the young tissue culture plants from the laboratory revealed expression of *gus*A in all roots of RCP-1:GUS lines and CaMV35S:GUS lines. The CaMV35S:GUS lines had uniform blue staining in leaves (Fig. 4a) and roots (Fig. 4b) whereas RCP-1:GUS lines stained intensely blue at the root tips and showed less intensity of blue coloration in the upper parts of the root (Fig. 4d). No staining was observed in the leaves of RCP-1:GUS lines (Fig. 4e). Similar results were observed for all of the 20 lines of RCP-1:GUS and the 15 lines of CaMV35S:GUS.

### Histochemical GUS analysis in infected roots of plantain

**Fig. 5:**
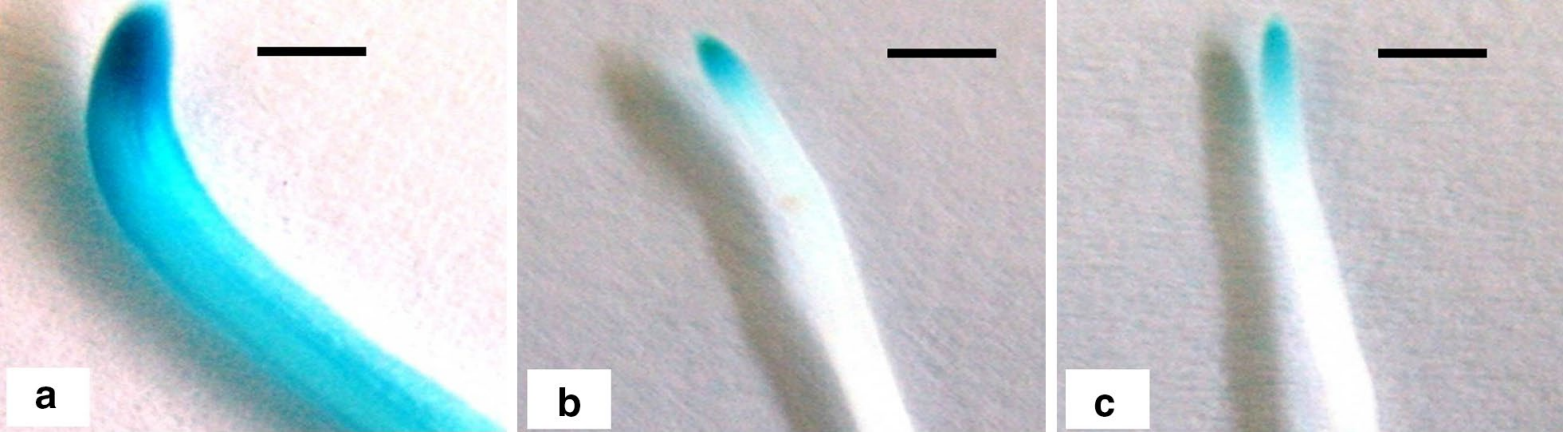
Histochemical GUS assay performed on nematode infected roots of transgenic plantains at 28 days post infection. **a** GUS stained nematode infected root of 35S:GUS line. **b** GUS stained nematode infected root of RCP-1:GUS line. **c** GUS stained uninfected root of RCP-1:GUS line *Scale bar* 5 mm

The GUS activity in plants grown in soil with or without nematode challenge was assessed. The pot grown CaMV35S:GUS lines showed uniform expression of *gus*A in roots at 28 days post infection (dpi) (Fig. 5a) similar to in vitro plants of the same line (Fig. 4). The same was true for the pot grown RCP1:GUS lines, which showed *gus*A expression only in the root tips at 28 dpi with *R. similis* (Fig. 5b) similar to in vitro plants of the same line (Fig. 4). Infection with *R. similis* had no effect upon the *gus*A expression pattern of RCP-1:GUS lines (Fig. 5b) when compared to non-infected plants (Fig. 5c).

### Nematode root invasion and establishment

**Fig. 6:**
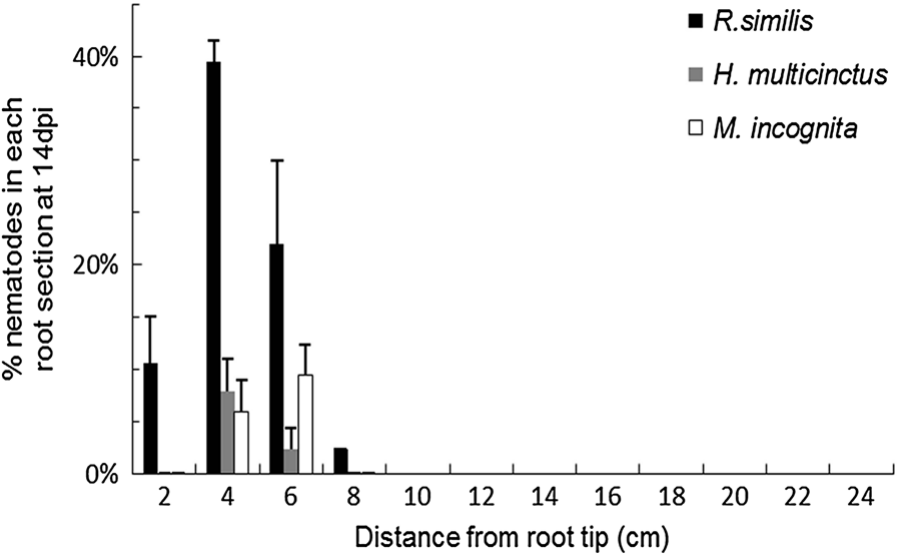
Root invasion assay for nematode infection. Establishment in root tips of ‘Gonja manjaya’ at 14 days post infection by *Radopholus similis*, *Helicotylenchus multicinctus* and *Meloidogyne incognita*

Two experiments, using two different cultivars of banana, were carried out to study root invasion by nematodes. In a preliminary experiment, which was done to establish the protocol with banana cv. ‘Cavendish’, 100 *R. similis* were inoculated close to root tips of 16 non-transgenic banana plantlets of cv. ‘Cavendish’; all nematodes were within 15 cm of the root tip. The distribution of the nematodes was such that 50 % of them (±95 % confidence limits) were present within 5.92 ± 0.77 cm of the root tips for the ‘Cavendish’ plants. This was followed by an experiment with the transgenic cv. ‘Gonja manjaya’ plants developed in this study along with non-transgenic controls. A mixed population of *R. similis*, *Helicotylenchusmulticinctus* and *M.incognita* was used for inoculation of ‘Gonja manjaya’. The population of *R. similis* was highest near the root tips of this plantain and declined distally. At 7 dpi, 83 % of *R. similis* were present in the first 8 cm distal to the root tip but a few nematodes were also present at 12 and 24 cm. However, at 14 dpi all *R. similis* were within 8 cm of the root tip (Fig. 6). In total, only eight *M. incognita* and eight *H. multicinctus* were recovered from the roots and all were within 6–12 cm of the root tip (Fig. 6).

## Discussion

### Analysis of GUS activity in transgenic plantains

This work establishes the potential of ZmRCP-1 promoter isolated from maize for delivering transgenic traits when activity is only required at the root tips of transgenic plantain. As expected, the CaMV35S promoter provided intense *gus*A expression along the root and in leaf tissues (Figs. 4, 5). Such constitutive expression is an undesirable trait in commercial transgenic plants when only particular tissues need to express a transgene to provide the trait of interest such as nematode resistance. ZmRCP-1 has provided an expression pattern that is limited to the root tips of both tissue culture and soil grown plants up to 28 dpi (Fig. 5). Further work is required to establish the root expression pattern across the whole crop cycle under field conditions. There is a need to confirm the expression in younger root tissues close to the tips persists in mature plants and that the nematodes remain predominately in this region. Additionally, protection of roots in younger banana plants is important to favour crop establishment.

In maize, ZmRCP-1 expression is restricted to the lateral root cap cells and does not extend more than about 0.3 mm behind the root tip [24]. In contrast, this promoter delivered expression of *gus*A gene in transgenic plantain that extended throughout young roots and to 0.5 cm from the tip in mature roots (Fig. 6). A similar difference in pattern of expression between plants has been reported for MDK4-20 a homolog of ZmRCP-1 isolated from *A. thaliana* [13]. The MDK4-20 promoter directed the expression of *gus*A gene specifically to peripheral cells of root tip and lateral root cap cells of *A. thaliana* [13, 24]. Its activity in potato was also in outer cells of the root tip but it extended further towards the zone of elongation. This less restricted pattern of expression in potato delivered a higher level of repellent peptide defence to cyst nematodes in potato than in *A. thaliana* where the pattern of expression was more limited [13].

In this study, vegetatively micro-propagated transgenic plantains of cv. ‘Gonja manjaya’ for several cycles were used to investigate the *gus*A expression pattern under the control of ZmRCP-1 promoter. Similar study has been conducted with vegetatively micro-propagated *S. tuberosum* to establish the root cap specific expression of *gus*A under AtMDK4-20 promoter [13]. Transgenic plants of ‘Gonja manjaya’ expressing a cystatin and a synthetic peptide to provide single and dual resistance against soil nematodes have also been studied under screen house and field conditions [8, 12]. Field trial of *Xanthomonas* wilt disease-resistant bananas have been carried out using vegetatively propagated bananas [25]. In other studies, vegetatively micro-propagated plants have been used to confirm the role of *A. thaliana* NHL1 and NHL8 genes in the Soybean defence mechanism against *Heterodera glycines* [26] and in post-transcriptional hairpin RNA-mediated gene silencing of vital fungal genes to confer resistance against *Fusarium* wilt in banana [27].

### The GUS activity in nematode infected roots

There were no significant differences between the population means of *R. similis* per 100 g root for the transgenic plants of ZmRCP-1 lines and CaMV35S lines and non-transgenic control plants. The *gus*A gene expression is known not to affect nematode establishment in plants and this has been evident in previous studies of nematode invasion [19, 21]. A decline in ZmRCP-1 promoter activity with root age is also evident in this study (Figs. 4, 5). The GUS activity becomes root cap specific and the promoter activity is restricted within 5 mm from the tip at 28 dpi (Fig. 5). In younger roots, GUS activity was high at the root tip but also extended further up the root end (Fig. 4). It is observable that there was a variation in the intensity of GUS expression between the roots of young tissue culture plants and those of older plants at 28 dpi (Figs. 4, 5). A decline in promoter activity has been reported previously for other root active promoters. For instance it occurs partially for that from ubiquitin-1 (Ubi-1) and fully for the promoter of tubulin-1 (Tub-1) over 10 weeks in rice roots [19]. On rice, *Pratylenchus zeae* feeds similarly to *R. similis* on *Musa* spp. Similar to this work, nematodes did not modify Ubi-1 or Tub-1 promoter activity in rice [19].

The particular advantage of expressing anti-nematode defences in root cap cells is that they are responsible for much of root exudation. They also remain active for a period of up to a few days after detachment from the root [13]. This ensures delivery of the defence to the nematode in the rhizosphere before root invasion can occur. The efficacy of a root-cap active promoter in delivering nematode control also depends on other factors. One is the rate of root border cell production. Two *Musa acuminata* cultivars produce a relatively high number of these cells [28] and many more than *Solanaceae* [13, 29]. Border cell production is conserved at the plant family level but regulated by endogenous and environmental signals [29].

### Nematodes invasion and establishment in the roots

This work established that *R. similis* preferentially feed in the first few centimeters of both plantain cv. ‘Gonja manjaya’ (Fig. 6) and dessert banana cv. ‘Cavendish’ roots. While expression from the ZmRCP-1 is present in the more proximal root regions that harboured nematodes, previous studies have reported a preferential invasion and establishment of nematodes close to the root tip. *M. incognita* [30] and *R. similis* [31, 32] preferentially invade the roots near the root apex. Expression at the root apex and in root border cells, as is the case here, therefore delivers the peptide to the locale of nematode invasion. Constitutive expression of the peptide-mediated nematode defence is not required in parts of the plant where these parasites do not occur. Restricting expression to roots avoids unwanted exposure to the transgene of non-target organisms associated with aerial tissues. It also ensures that the plant is not burdened with miss-targeted production of the defence.

## Conclusion

Targeted expression of a transgenic defence designed to disrupt nematode invasion and establishment requires a root tip specific promoter. Such promoters exist for dicot plants; for instance, phytase has been secreted into the rhizosphere of potato plants using the promoter of the tomato LeExt1.1 gene that directs expression in trichoblasts [33]. However, such promoters are not available for monocot plants. Our study demonstrated that ZmRCP-1 promoter directs the expression of *gus*A reporter gene to root cap in transgenic plantain. ZmRCP-1 promoter has potential to provide targeted expression of transgene for nematode resistance in transgenic plantain, potentially contributing to the biosafety of nematode resistant plantains if it delivers effective peptide-mediated resistance to banana nematodes throughout the cropping cycle. We intend to develop transgenic plantains expressing a repellent peptide under the control of ZmRCP-1 promoter.

## Methods

### Plasmid constructs

For the root cap specific construct, the ZmRCP-1 promoter was cloned from a Corn Lambda Genomic Library (Stratagene, La Jolla, CA, USA) using forward (5′-GTTACTAAGCTTCCTATGTCAATTAAGGGAGTTGATG-3′) and reverse (5′-GTTACTGGATCC-AGCTCATACTGCTTCTGTGACTGT-3′) primers that amplified 2 kbp immediately 5′ to the RCP-1 open reading frame [24] and introduced 5′ *Hin*dIII and 3′ *Bam*HI restriction sites. Amplification was performed using Phusion High-Fidelity DNA Polymerase (NEB, MA, USA) using cycling conditions of 30 s @ 98 °C followed by 30 cycles of 10 s @ 98 °C, 15 s @ 60 °C and 30 s @ 72 °C with a final extension step at 72 °C for 10 min. The ZmRCP-1 promoter fragment was cloned into the *Hin*dIII and *Bam*HI sites of the binary vector pBI121 [34] immediately 5′ to the *β*-*glucuronidase* (*gusA*) gene to generate construct pBI-RCP-1:GUS (Fig. 1a) using standard molecular cloning techniques [35]. For the constitutive expression construct, pBI121 plasmid that already contained the *gusA* gene under regulation of CaMV35S promoter [34] was used (Fig. 1b). Both constructs were confirmed by sequencing before transforming into *A. tumefaciens* strain EHA105 [36] by electroporation. Constructs were verified by PCR analysis using the same primers used for cloning the ZmRCP-1 promoter. For pBI121, forward primer (5′-ACATCTAGAATGGTGGAGCACGACAC-3′) and reverse primer (5′-ACAGGATCCTCGAGAGAGATAGATTTG-3′) were used that amplify the 835 bp of CaMV5S promoter (Fig. 1). Amplification conditions were the same as for cloning.

### Plant material, transformation and regeneration

Embryogenic cell suspension of plantain cv. ‘Gonja manjaya’ (*Musa* spp. AAB) was transformed as described previously [16]. The *Agrobacterium*-infected embryogenic cell suspensions were regenerated on selective medium supplemented with cefotaxime (300 mg L^−1^) and kanamycin (100 mg L^−1^) with transfer every 2 weeks to fresh medium of the same type. The regenerated transgenic shoots were maintained and multiplied on proliferation medium consisting of Murashige and Skoog (MS) medium [37] supplemented with 5 mg L^−1^ benzylaminopurine (BAP), at 28 °C for a 16/8 h light/dark photoperiod under fluorescent tube lights. Regenerated putative transgenic shoots were transferred to rooting medium (MS medium supplemented with 1 mg L^−1^ indole-3-butyric acid [IBA]). Rooted plantlets were transferred to sterile soil in pots and maintained in a contained glasshouse. Regenerated putative transgenic shoots were regularly micro-propagated and clonally multiplied to obtain sufficient plantlets of each line for nematode challenge in glasshouse and maintenance of the line in vitro.

The transformation experiments were performed at biosafety level II research facility of National Agricultural Research Laboratories following the national biosafety guidelines.

### Genomic DNA isolation and PCR analysis of transgenic lines

The plant genomic DNA was extracted from the regenerated putative transgenic plants using a DNeasy kit (Qiagen, Hilden, Germany) from leaf tissue of in vitro plants ground using liquid nitrogen. DNA quality and concentration was determined by NanoDrop 2000c (Thermo Scientific, Wilmington, USA). PCR analysis was performed using *gus*A gene specific primers to confirm the presence or absence of the transgene in the plant genome. The forward (5′-TTTAACTATGCCGGAATCCATCGC-3′) and reverse (5′-CCAGTCGAGCATCTCTTCAGCGTA-3′) primers amplify a 528 bp region of the *gus*A gene. PCR amplification was carried out in a total volume of 20 µL containing 4 µL of 200 ng µL^−1^ genomic DNA, 0.5 µL of each 0.2 μM primer and 10 µL of GoTaq Green Master Mix (Promega, Madison, USA). Thermocycling began with denaturation at 94 °C for 5 min, followed by 35 cycles of 94 °C for 50 s, 55 °C for 40 s and 72 °C for 50 s, and a final extension of 72 °C for 10 min.

### Histochemical detection of *gus*A expression in transgenic plantain

Histochemical detection of the *gus*A gene was carried out according to the modified protocol of Jefferson [38] as described in Tripathi et al. [16]. Roots and leaf material were washed in 70 % ethanol for 2 min and fixed in 0.3 % v/v formaldehyde, 10 mM MES, pH 5.6, 0.3 M mannitol for 45 min at room temperature followed by three washes in 50 mM sodium phosphate, pH 7.0.

The fixed material was vacuum-infiltrated in substrate solution (1 mM X-gluc, 50 mM sodium-phosphate, pH 7.0, 5 mM potassium ferricyanide, 5 mM potassium ferrocyanide, 10 mM EDTA and 50 mM ascorbic acid) for 4 min and then incubated at 37 °C for 48 h. The chlorophyll of the leaf material was then removed by immersing in 1 % NaOCl solution for 3 h and subsequently dehydrated in sequential 30 min incubations in 50, 70 and 95 % ethanol. Staining was imaged by DSC-F828 camera (Sony, New York, NY, USA).

### Nematode challenge

The nematode challenge trial was carried out in a contained screen house authorised for use with transgenic plants at the National Agricultural Research Laboratories (NARL) at Kawanda, Uganda. Forty two transgenic plants and six non-transgenic plants for the nematode challenge trial were set up in a randomized design within the screen house. Three plants each of five lines of ZmRCP-1, two lines of CaMV35S and non-transgenic control plants (24 plants in total) were challenged with nematodes in a bioassay. The potted plants were grown for 8 weeks before inoculation with 1000 juveniles of *R. similis* per plant. The nematodes were watered onto GF/A filter paper and placed onto exposed roots. Three plants of each transgenic and the non-transformed lines were left uninfected as controls. The trial continued for a further 4 weeks in the screen house at ambient temperatures with daily watering. The height, girth and numbers of functional leaves were recorded at 2 week intervals and were used to calculate the total leaf area (TLA), TLA = n (0.411G + 0.381H−0.404) where n is the number of functional leaves more than 50 % green and fully attached to the pseudostem, G is girth (cm) at the base of the pseudostem and H is the plant height (cm) measured from the base to the axil of the topmost pair of fully expanded leaves [39]. Root samples, including root tip, were collected from each plant at infection and at 4 weeks for histochemical detection of *gus*A expression. At the end of the trial, the root tissue fresh weight was recorded before the roots were cut into 1 cm pieces. Root samples were placed in polypropylene bags and submerged in 10 ml of 1 % H_2_O_2_ solution [40]. Nematodes were collected and counted using a stereo-microscope after 7 days of incubation at room temperature in the dark.

### Analysis of roots invasion by nematodes

The analysis was done to determine the distribution of nematodes population at various root sections from the start of the infection period. An initial experiment was carried out using 16 plants of banana cv. ‘Cavendish’ that were infected with *R. similis* for 28 days to determine the distribution of nematodes along the length of roots in which the nematodes occurred. The subsequent experiment used mixed population of 1000 nematodes containing equal numbers of *R. similis*, *H. multicinctus* and *M. incognita* to infect roots of 10 non-transgenic control plants of cv. ‘Gonja manjaya’. At 7 days post infection (dpi), roots of five of the plants were collected cut into 2 cm sections and pooled into 2 cm groups from the root tip. The nematodes that emerged from the roots were counted in 200 µl aliquots using a stereo-microscope. The procedure was repeated for the remaining five plants at 14 dpi.

### Statistical analysis

All data were analyzed using SPSS v20 (IBM Corporation Armonk, New York, USA; http://www-01.ibm.com/software/analytics/spss). The choice of analysis used for data was informed by both the help files of the package and a standard text [41]. A univariate ANOVA was carried out to establish any significant differences in the growth parameters of infected ZmRCP-1 and CaMV35S cell lines. Levene’s test was used to establish homogeneity of variances.
